# Supplementary material for: Long-term safety and efficacy of ferric citrate in phosphate-lowering and iron-repletion effects among patients with on hemodialysis: A multicenter, open-label, Phase IV trial
Source: PLoS One. 2022 Mar 3;17(3):e0264727. doi: 10.1371/journal.pone.0264727 (PMC8893642; doi:10.1371/journal.pone.0264727)
Supplement: S1 Table — (DOCX) [file pone.0264727.s003.docx]

**S1 Table**. Changes of Clinical Laboratory Parameters During the Treatment Period (N=202)

|  | M0 | EOT |
| --- | --- | --- |
| Aluminum (nmol/L) | 515.90 ± 476.94 | 485.40 ± 328.72 |
| Sodium (mmol/L) | 137.97 ± 2.71 | 137.55 ± 2.86 |
| Potassium (mmol/L) | 4.68 ± 0.66 | 4.56 ± 0.70 |
| Albumin (g/L) | 40.13 ± 3.04 | 39.69 ± 3.61 |
| ALT (U/L) | 13.99 ± 6.89 | 14.20 ± 7.80 |
| AST (U/L) | 16.61 ± 6.65 | 17.88 ± 13.39 |

Data was presented as mean ± standard deviation.

Abbreviations: M0, baseline; EOT, end of treatment; ALT: alanine aminotransferase; AST: aspartate aminotransferase.
