# Supplementary material for: Long-term safety and efficacy of ferric citrate in phosphate-lowering and iron-repletion effects among patients with on hemodialysis: A multicenter, open-label, Phase IV trial
Source: PLoS One. 2022 Mar 3;17(3):e0264727. doi: 10.1371/journal.pone.0264727 (PMC8893642; doi:10.1371/journal.pone.0264727)
Supplement: S2 Table — (DOCX) [file pone.0264727.s004.docx]

**S2 Table**. Most Common Drug-related TEAEs with Incidence Rate > 1% (N=202)

| Drug-related TEAEs by SOC and PT | | N | (%) |
| --- | --- | --- | --- |
| Gastrointestinal disorders | |  |  |
|  | Discolored feces | 83 | (41.1%) |
|  | Diarrhea | 21 | (10.4%) |
|  | Constipation | 16 | (7.9%) |
|  | Abdominal distension | 8 | (4.0%) |
|  | Abdominal pain | 8 | (4.0%) |
|  | Soft feces | 5 | (2.5%) |
|  | Abdominal pain upper | 3 | (1.5%) |
| Metabolism and nutrition disorders | |  |  |
|  | hyperferritinemia | 8 | (4.0%) |
| Skin and subcutaneous tissue disorders | |  |  |
|  | Pruritus | 6 | (3.0%) |

Data was presented as number and percentage, and the N was based on the number of patients experiencing ≥ 1 TEAE, not the number of events.

Abbreviations: SOC, system organ class; PT, preferred term.
