# Supplementary material for: Long-term safety and efficacy of ferric citrate in phosphate-lowering and iron-repletion effects among patients with on hemodialysis: A multicenter, open-label, Phase IV trial
Source: PLoS One. 2022 Mar 3;17(3):e0264727. doi: 10.1371/journal.pone.0264727 (PMC8893642; doi:10.1371/journal.pone.0264727)
Supplement: S3 Table — (DOCX) [file pone.0264727.s005.docx]

**S3 Table**. Most Common TEAEs Leading to Drug Discontinuation with Incidence Rate >1% (N=202)

| TEAEs by PT | | N | (%) |
| --- | --- | --- | --- |
|  | Diarrhea | 7 | (3.5%) |
|  | Abdominal distension | 4 | (2.0%) |
|  | Pruritus | 4 | (2.0%) |
|  | Abdominal pain | 3 | (1.5%) |
|  | Constipation | 3 | (1.5%) |
|  | Rash | 3 | (1.5%) |

Data was presented as number and percentage, and the N was based on the number of patients experiencing ≥ 1 TEAE, not the number of events.

Abbreviations: PT, preferred term.
