# Supplementary material for: Long-term safety and efficacy of ferric citrate in phosphate-lowering and iron-repletion effects among patients with on hemodialysis: A multicenter, open-label, Phase IV trial
Source: PLoS One. 2022 Mar 3;17(3):e0264727. doi: 10.1371/journal.pone.0264727 (PMC8893642; doi:10.1371/journal.pone.0264727)
Supplement: S4 Table — (DOCX) [file pone.0264727.s006.docx]

**S4 Table. Changes of iPTH levels after stratified by concomitant vitamin D treatment** (N=197)

|  | | **M0** | **EOT** | **Change from**  **M0 to EOT** | **P-value** |
| --- | --- | --- | --- | --- | --- |
| **Total ESRD patients (N=197)** | | 453.25 ± 32.36 | 555.37 ± 35.41 | 101.35 ± 26.37 | <0.001 |
| **Concomitant vitamin D** | **No**  **(N=99)** | 323.82 ± 40.98 | 472.94 ± 47.74 | 149.76 ± 31.94 | <0.001 |
|  | **Yes**  **(N=98)** | 584.00 ± 46.79 | 635.02 ± 51.00 | 54.59 ± 41.23 | 0.189 |

Data was presented as mean ± standard error. The paired sample t-test was used to compare two means between M0 and EOT.

Abbreviations: iPTH, intact parathyroid hormone; ESRD, end-stage renal disease; M0, baseline; EOT, end of treatment.
