# Supplementary figures and images for: Long-term safety and efficacy of ferric citrate in phosphate-lowering and iron-repletion effects among patients with on hemodialysis: A multicenter, open-label, Phase IV trial
Source: PLoS One. 2022 Mar 3;17(3):e0264727. doi: 10.1371/journal.pone.0264727 (PMC8893642; doi:10.1371/journal.pone.0264727)

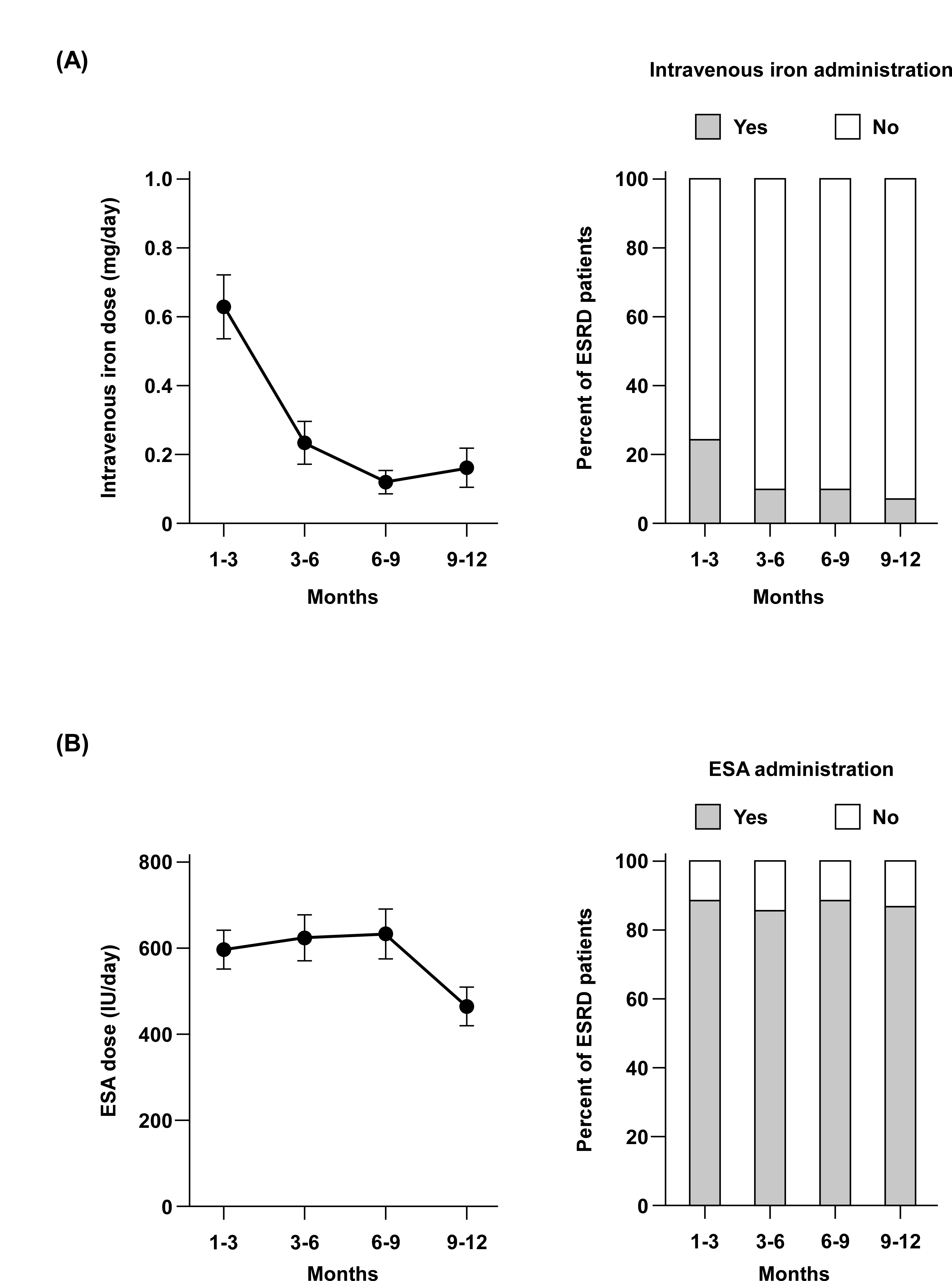

Supplement: S1 Fig — (A) Intravenous iron dosage and administration. (B) ESA dosage and use. (JPG) [file pone.0264727.s007.jpg]
