# Supplementary material for: Long-term safety and efficacy of ferric citrate in phosphate-lowering and iron-repletion effects among patients with on hemodialysis: A multicenter, open-label, Phase IV trial
Source: PLoS One. 2022 Mar 3;17(3):e0264727. doi: 10.1371/journal.pone.0264727 (PMC8893642; doi:10.1371/journal.pone.0264727)
Supplement: S1 Appendix — (PDF) [file pone.0264727.s008.pdf]

CLINICAL STUDY PROTOCOL

STUDY TITLE: **A Long-Term, Open-Label, Prospective Observational Phase IV Study to Assess the Safety and Efficacy of ferric citrate (Nephoxil®) in Subjects with End Stage Renal Disease (ESRD) on Dialysis**

PROTOCOL NUMBER: Protocol # PBB00501

DEVELOPMENT PHASE: IV

STUDY DRUG: Nephoxil® Capsule (ferric citrate, code: PBF-1681)

INDICATION: Hyperphosphatemia in End Stage Renal Disease Patients

Chien-Te Lee, MD, PhD[1], Ching-Chang Lee, MD[2], Ming-Ju Wu, MD, PhD[3], Yi-Wen Chiu, MD[4], Jyh-Gang Leu, MD, PhD[5], Ming-Shiou Wu, MD, PhD[6], Yu-Sen Peng, MD, PhD[7], Mai-Szu Wu, MD[8, 9, 10], Der-Cherng Tarng, MD, PhD[11]

1. Division of Nephrology, Department of Internal Medicine, Kaohsiung Chang-Gung Memorial Hospital and Chang Gung University College of Medicine, Taiwan.
2. Division of Nephrology, Department of Internal Medicine, Keelung Chang-Gung Memorial Hospital, Taiwan.
3. Division of Nephrology, Department of Internal Medicine, Taichung Veterans General Hospital, Taiwan.
4. Division of Nephrology, Department of Internal Medicine, Kaohsiung Medical University Chung-Ho Memorial Hospital, Taiwan.
5. Division of Nephrology, Department of Internal Medicine, Shin Kong Wu Ho-Su Memorial Hospital, Taiwan.
6. Division of Nephrology, Department of Internal Medicine, National Taiwan University Hospital, Taiwan.
7. Division of Nephrology, Department of Internal Medicine, Far-Eastern Memorial Hospital, Taiwan.
8. Division of Nephrology, Department of Internal Medicine, Shuang Ho Hospital, Taipei Medical University, Taiwan.
9. Department of Internal Medicine, School of Medicine, College of Medicine, Taipei Medical University, Taiwan.
10. TMU Research Center of Urology and Kidney, Taipei Medical University, Taipei, Taiwan.
11. Division of Nephrology, Department of Medicine, Taipei Veterans General Hospital, Taiwan.

Correspondence to:
Mai-Szu Wu, MD/ E-mail: maiszuwu@gmail.com
Der-Cherng Tarng, MD, PhD/ E-mail: dctarng@vghtpe.gov.tw

TABLE OF CONTENTS

TABLE OF CONTENTS 2
1 STUDY SYNOPSIS 3
2 INTRODUCTION 6
3 STUDY OBJECTIVES 7
4 STUDY VARIABLES 7
4.1 Variables to Determine the Safety 7
4.2 Variables to Determine the Efficacy 8
4.3 Variables for Exploratory Endpoints 8
4.4 Other Assessment Variables 8
5 STUDY DESIGN 8
5.1 Study Description 8
5.2 Planned Number of Subjects and Sites 9
6 SELECTION AND WITHDRAWAL OF SUBJECTS 9
6.1 Inclusion Criteria 9
6.2 Exclusion Criteria 9
6.3 Withdrawal Criteria 10
7 STUDY MEDICATIONS 10
7.1 Initial Dose 10
7.2 Dose Adjustments 10
7.3 Drug Accountability 10
7.4 Prior and concomitant medication / treatments 11
7.5 Prohibitions and Precautions 11
7.6 Blinding 11
8 STUDY PROCEDURES BY VISIT 11
8.1 Visit 1: Enrollment and Baseline Visit 11
8.2 Visit 2 to Visit 12 and Visit 14 11
8.3 Visit 13: End-of-Treatment /Early Termination Visit 12
9 STATISTICS 12
9.1 Determination of Sample Size 12
9.2 Definition of Analysis Sets 12
9.3 General Statistical Considerations 12
9.4 Analysis of endpoint 12
9.7 Handling of Missing Data 13
10 ASSESSMENT OF SAFETY 13
10.1 Adverse Events 13
10.2 Serious Adverse Events 14
10.3 Pregnancy 15
11 STUDY MANAGEMENT AND ADMINISTRATION 15
11.1 Adherence to Protocol 15
11.2 Monitoring 15
11.3 Data Handling 16
11.4 Good Clinical Practice 16
12 ETHICS AND REGULATORY REQUIREMENTS 16
12.1 Informed Consent 16
12.2 Institutional Review Boards and Independent Ethics Committees 16
12.3 Subject Confidentiality 17
12.4 Protocol Amendments 17
13 FINANCE, INSURANCE, AND PUBLICATION 17
14 REFERENCES 17

1 STUDY SYNOPSIS

Item	Description
**Study Title**	A Long-Term, Open-Label, Prospective, Observational Phase IV Study to Assess the Safety and Efficacy of ferric citrate (Nephoxil®) in Subjects with End Stage Renal Disease (ESRD) on Dialysis.
**Project Phase**	IV
**Study Objectives:**	To assess the long-term safety and effectiveness of ferric citrate for the treatment of hyperphosphatemia in patients with ESRD undergoing dialysis in the real world situation
**Study Design**	This study is an open-label, prospective, long term, observational Phase IV study to assess the safety and efficacy of ferric citrate in subjects with ESRD on dialysis.<br><br>Subjects who meet the eligibility criteria and provided informed consent will be enrolled to the observational study for up to 12 months of treatment with ferric citrate. No wash-out period will be required despite prior use of any oral phosphate binders.<br><br>Throughout the duration of the observation, study drug will be allowed for dose titration, targeting individual subject's serum phosphorus levels in between 3.5 and 5.5 mg/dL. Examinations to collect safety and efficacy measurements will be performed primarily according to routine hospital practice, except study drugs and additional tests that are specifically required to the study will be provided by the Sponsor.<br><br>The key measures collected for the patients will include, but not limited to, demography, medical history, physical examinations, vital signs, 12-lead electrocardiograms (ECG), clinical laboratory tests and prior/concomitant medication use for the evaluation of adverse events and treatment effectiveness throughout the observation period.
**Study Duration**	Each subject will participate in the study for approximately 13 months (including the enrollment visit/baseline visit, 12 routine monthly visits during treatment period for 12 months, and 1 follow-up visit after 1 month of the end of treatment.).<br>It is presumed the study will include a 3 months enrollment and a further 13 months to complete the follow-up for all enrolled patients.
**Planned Sample Size/ Study Center**	It is planned to recruit 200 patients in order to complete 13 months of observation for at least 100 subjects at 5~10 centers in Taiwan
**Patient Eligibility**	**Inclusion Criteria:**<br>A subject can participate in the study only if all the following criteria are met:<br>1) Is ≥ 18 years of age on the day of signing informed consent or other age required by local regulation<br>2) Willing and able to provide written informed consent<br>3) ESRD patients who is undergoing hemodialysis 3 times per week and is considered necessary to receive medication for hyperphosphatemia by his/her treating physician<br>4) Serum ferritin <1000 ng/mL and transferrin saturation (TSAT) < 50% at the Enrollment Visit<br>5) Women of child-bearing potential (WOCBP [defined as women ≤ 50 years of age with a history of amenorrhea for < 12 months prior to study entry]) who is willing to use an effective form of contraception during study participation.<br>**Exclusion Criteria:**<br>Subjects who meet any of the following criteria are not eligible to enter the study:<br>1) Has any known contraindication to ferric citrate according to locally approved prescribing information, include but not limited to the following criteria:<br>i. Is allergic to ferric citrate<br>ii. Has hypophosphatemia<br>iii. Has hemochromatosis or iron overload syndromes<br>iv. Has active severe GI disorders<br>2) Has parathyroidectomy (PTx) or percutaneous ethanol injection therapy (PEIT) within 3 months prior to Enrollment Visit or serum calcium < 7 mg/dL at the Enrollment Visit<br>3) Has participated in another interventional study for any investigational agent or device within 30 days prior to enrollment

Item	Description
	4) Is currently pregnant or breastfeeding<br>5) Other unstable medical condition or psychiatric conditions that is considered unsuitable for this study per Investigator's clinical judgment<br><br>**Withdrawal criteria:**<br>A subject may be removed from the study at any time if any of the following criteria is met:<br>• Subject request<br>• Treatment failure (see below)<br>• Investigator's discretion for the best interest of subject<br>• Intercurrent illness or medical event necessitating study drug discontinuation<br><br>If a subject has serum phosphorous level >8.0 mg/dL in two consecutive monthly visits, not due to subject-reported non-compliance, the subject will be considered a treatment failure and will discontinue from the study.
**Test product, dose and mode of administration**	Nephoxil® Capsules 500 mg (Ferric citrate)<br><br>All subjects will be instructed to take study drug intact with meals or immediately after meals and should not be opened or grounded.<br><br>Dose administration for the study drug will be at the discretion of the treating physicians and according to individual subject's clinical condition. The initial dose and subsequent adjustments stated herein are to be considered as guidance for dose administration throughout the observation period, instead of mandated procedures.<br><br>If the subject was on prior phosphate binders at a dosage equivalent to < 4.5g/day of calcium-based phosphate binders before entering the study, the suggested initial dose of study drug will be started from 3 g/day (2 capsules/meal, 6 capsules/ day).<br><br>If the subject was on prior phosphate binders at a dosage equivalent to ≥ 4.5 g/day of calcium-based phosphate binders before entering the study, the suggested initial dose of study drug will be started from 4.5 g/day (3 capsules/meal, 9 capsules/ day).<br><br>The dose will be adjusted 1-2 g/day to meet target goal for serum phosphorus of 3.5 to 5.5 mg/dL with a maximum dose of 12 g/ day.
**Prior and concomitant medication / treatments**	Specific prior medication/treatments, including oral phosphate binders, ESA, iron preparation, aluminum containing medication, Vitamin D & calcium supplements, anti-osteoporotic medications, or other drugs that affect serum phosphorus level, during the past 3 months prior to study enrollment which meet the criteria below are considered relevant to the study indication and should be collected.<br><br>Any concomitant medication taken in addition to the study treatments and is considered relevant to influence serum phosphorous and/or iron levels should be collected throughout the study.<br><br>Other concomitant medications taken, only minimal information will be collected throughout the study.
**Main Parameters of Evaluation**	**Primary Endpoints (Safety assessment):**<br>• To assess the safety profiles from baseline to the end-of-treatment.<br>Safety profiles are assessed by monitoring and recording the incidence of treatment-emergent adverse events (TEAEs) and serious TEAEs whether related to study treatment or not, as determined by clinically significant changes in laboratory tests (i.e., hematological tests and biochemical tests, including iron panels), physical examinations, vital signs, standard 12-lead electrocardiogram and any other untoward medical events during the study period as judged by Investigator. All subjects who receive at least one dose of study medication will be evaluated for safety.<br><br>**Secondary Endpoints (Efficacy assessment)**<br>To evaluate serum phosphorus by the time-course changes of serum phosphorus levels and

Item	Description
	proportion of subjects achieved the target range of serum phosphorus (≥3.5 mg/dL and ≤ 5.5 mg/dL).<br><br>**Exploratory Endpoints**<br>The following parameters will be explored:<br>• Time-course change of serum calcium and intact plasma parathyroid hormone (iPTH)<br>• Time-course changes of iron-parameters<br>• Time-course changes of hemoglobin<br>• Changes in dose of IV iron and ESA<br>• Treatment adherence
**Statistical Analysis**	**Safety assessment:**<br>Treatment-emergent adverse events (TEAEs) will be summarized by system organ class, preferred term, severity, and suspected relationship to study drug. Clinical laboratory tests and vital signs of observed values and changes from baseline will be summarized using descriptive statistics. Physical examination findings and ECGs will also be summarized descriptively.<br><br>**Efficacy assessment**<br>• The mean and the 95% confidence interval for the mean values are to be calculated at for each observation day and at the EOT.<br>• Proportion of subjects achieved the target range of serum phosphorus (≥3.5 mg/dL and ≤ 5.5 mg/dL) from baseline to the end-of-treatment will be tabulated or plotted, as appropriate.<br>**Exploratory analyses**<br>• Time-course change of serum calcium and iPTH: Changes in serum calcium and iPTH will be summarized descriptively as observed values and as changes from baseline<br>• Time-course changes of iron-parameters: Changes of iron parameters will be summarized descriptively as observed values and as changes from baseline<br>• Time-course changes of hemoglobin: Changes in hemoglobin will be summarized descriptively as observed values and as changes from baseline<br>• Change in dose of IV iron and ESA: Changes in dose of IV iron and ESA from baseline to EOT will be summarized descriptively.<br>• Treatment adherence: Descriptive statistics of the prescribed dose and the dose actually taken at each time point will be calculated.

2 INTRODUCTION

In normal individuals, phosphate is primarily eliminated by the kidneys which effectively regulate phosphate balance and blood phosphate concentration. Thus, in patients with end stage renal disease (ESRD) there is a marked decrease in phosphorus excretion in the urine, leading to hyperphosphatemia[1] unless they decrease their phosphate intake or treated with phosphate-binding agents to prevent absorption. There are serious long-term consequences of hyperphosphatemia, including bone demineralization, nausea and weakness[2]. In response to hyperphosphatemia, there is increased production of parathyroid hormone (PTH), a condition known as secondary hyperparathyroidism. If not treated, secondary hyperparathyroidism may cause many adverse clinical outcomes, one of which is a form of bone disease referred to as renal osteodystrophy, which can lead to pain and may progress to pathological fractures and significant skeletal deformity. Secondary and subsequent tertiary hyperparathyroidism, hyperphosphatemia, and elevated calcium phosphate have also been associated with increased mortality and other non-bone-related sequelae associated with extra-osseous calcification. It has been shown that a high intake of dietary phosphorus in experimental renal failure worsens renal function [3, 4] and low phosphate intake arrests the progression of chronic renal failure [2-4]. Restriction of dietary phosphate is usually not sufficient to reverse the hyperphosphatemia in patients with ESRD, therefore these patients are traditionally treated with orally administered aluminum or calcium compounds that bind dietary phosphate and facilitate fecal elimination rather than intestinal absorption. A variety of phosphate-binding agents have been used clinically including aluminum salts, calcium salts, sevelamer hydrochloride or carbonate, and lanthanum carbonate. These agents are effective in phosphate binding, however, there are specific safety, tolerability or cost concerns with each. Long-term administration of aluminum salts is associated with bone toxicity (it appears to block calcium deposition in the bone matrix and may interfere with osteoblast function as well [5, 6]) damage to neurological functions, and other toxicities (it accumulates in the parathyroid glands and may interfere with the secretion of parathyroid hormone [PTH], contributing to low bone-turnover)[5-7]. This has led to calcium salts being the phosphate-binding agents of choice. However, use of such compounds can lead to hypercalcemia, with resulting metastatic calcification and organ dysfunction [8-12]. In the ESRD patient, this can be further compounded by vitamin D therapy, lack of a reliable rout of calcium excretion, hyperparathyroidism, and calcium-containing dialysate.

Nephoxil (ferric citrate) is an oral, non-calcium iron-based phosphate-binding agent that was approved to the control of serum phosphorus levels in patients with chronic kidney disease on dialysis. The active ingredient of Nephoxil® capsule is pharmaceutical grade ferric citrate which has been approved as a phosphate binder in Taiwan, United States, Japan and European Union. The ferric iron from ferric citrate reacts with the phosphorus in the GI tract, precipitating phosphorus as ferric phosphate. The latter is insoluble and is excreted in the stool. This process inhibits the gastro-intestinal absorption of phosphate and reduces its systemic availability thus reducing serum phosphorus level.

Previous clinical studies have demonstrated the ability of ferric citrate to manage serum phosphorus in subjects with ESRD on dialysis. In the Taiwan pivotal Study, which is a randomized, double-blind, fixed dose, placebo-controlled study, serum phosphorus shows notable decrease as soon as receiving 1 week of treatment in both 4 g/day and 6 g/day groups. At the end of the study, the mean decrease from baseline in serum phosphorus is from 6.95 to 4.69 mg/dL (-2.27 mg/dL) in the 6 g/day group, and 6.96 to 5.38 mg/dL (-1.60 mg/dL) in 4 g/day group. Both dose groups achieved statistical significance of P-value <0.001.

Ferric citrate has been administered to the ESRD patients from 1 g/day to up to 12 g/day, and the study duration was from 4 weeks to at least 52 weeks safety monitoring in previous clinical

programs. Across these clinical studies, ferric citrate demonstrated a consistent AE profile and safety profile. Based on the clinical experiences, the adverse events of ferric citrate are mostly associated to mild to moderate GI tract discomfort and among which discolored feces is the most common adverse reaction reported, followed by constipation, abdominal distension, diarrhea and abdominal pain. Although clinical studies conducted todate have demonstrated that ferric citrate has an acceptable risk-benefit profile, there is a need for information from real-world clinical practice to establish the long-term safety profile of ferric citrate in Asian/Chinese population.

3 STUDY OBJECTIVES

The objectives of this study are to assess the long-term safety and effectiveness of ferric citrate for the treatment of hyperphosphatemia in patients with ESRD undergoing dialysis in the real world situation.

4 STUDY VARIABLES

4.1 Variables to Determine the Safety

The safety assessment is to assess the safety profiles from baseline to the end-of-treatment (EOT). Safety profiles will be assessed by monitoring and recording the incidence of treatment-emergent adverse events (TEAEs) and serious TEAEs, whether related to study treatment or not, as determined by changes in clinical laboratory tests (i.e., hematological tests and biochemical tests, including iron panels), physical examinations, vital signs, standard 12-lead electrocardiogram and any other untoward medical events during the study period. All subjects who receive at least one dose of study medication will be evaluated for safety.

To ensure complete safety data collection, all AEs occurring after signing the Informed Consent form, including any pre-treatment and post-treatment periods required by the protocol, shall be recorded. TEAEs will be defined as adverse events (AEs) with an onset date on or after the first dose of study treatment. AEs with missing onset dates will be assumed to be treatment-emergent. The incidence of TEAEs (any TEAE, serious TEAEs, drug-related TEAEs, serious drug-related TEAEs, and TEAEs leading to treatment discontinuation, or death) will be summarized, respectively. If a TEAE is reported more than once by a subject during the study, the one with the greatest severity will be included in the summary tables. A TEAE will be considered drug-related if the Investigator indicates that the event is “possibly”, “probably”, or “definitely” related or if the relationship is missing.

The variables used to describe TEAEs are listed below:

1) Clinical laboratory tests will be recorded at every visit including essential examinations and optional examinations. Essential examinations consist of routine hospital examinations and study-mandatory examinations. The essential examinations will be performed and collected according to protocol schedule at every visit unless otherwise stated (i.e., serum aluminum performed at Visit 1 and EOT [Visit 13 or early termination]; iPTH performed at Visit 1, Visit 4, Visit 7, Visit 10, and EOT [Visit 13 or early termination]). Optional examinations, although not mandated, will be collected and assessed if results are available following routine practice.
2) Physical examination of all body systems will be performed at each visit including the Early Termination visit. Physical examination at Visit 1 will include measurement of height (cm/inches) without shoes. If any of the physical examination results at any visit is a clinically significant change from the first physical examination determined by the Investigator, that result should be documented as an AE.
3) Pulse rate (bpm), supine blood pressure (mmHg), body temperature and body weight are measured at all clinic visits.

4) Standard 12-lead ECGs will be taken at Baseline (Visit 1), and at Month 12 (Visit 13) or at the Early Termination visit. The ECGs will be read locally by the Investigator or qualified designated reader (signed/dated). The Investigator will confirm the computerized measurements of PR, QRS, QT, and QTcB intervals. Changes in ECG parameters will be relative to the measurements taken from the baseline visit (Visit 1) ECG. Consideration should also be given to performing an ECG if the subject should report any symptoms suspicious for cardiac events.
5) Any other treatment-emergent adverse events, including worsening of baseline diseases, will also be assessed at all clinic visits.

4.2 Variables to Determine the Efficacy

The efficacy assessment are to evaluate efficacy on serum phosphorus by assess of the time-course changes of serum phosphorus levels from baseline to the end-of-treatment and proportion of subjects achieved the target range of serum phosphorus (≥3.5 mg/dL and ≤ 5.5 mg/dL) from baseline to the end-of-treatment

4.3 Variables for Exploratory Endpoints

The following parameters will be explored:

- Time-course changes of serum calcium and intact plasma parathyroid hormone (iPTH) level from baseline to EOT.
- Time-course changes of Iron-parameters from baseline to EOT
- Time-course changes of hemoglobin from baseline to EOT
- Changes in dose of IV iron and ESA.
- Treatment adherence

4.4 Other Assessment Variables

Demographic data (age, gender, height, weight and race) will be recorded at Visit 1 (Baseline visit).

Medical and concurrent conditions at the time of initiation will be recorded in the medical and procedure history part of CRF at Visit 1 (Baseline visit) including

- ➢ Diagnosis date of CKD
- ➢ History of hemodialysis at the earliest known data point
- ➢ Primary etiology including
  - Diabetes nephropathy
  - Hypertensive nephrosclerosis
  - Non-diabetic glomerular disease (e.g. Focal segmental glomerulosclerosis (FSGS), membrane)
  - Polycystic kidney disease
  - Tubulointerstitial disease
  - Others
- ➢ Any previous or ongoing medical history and procedures or surgeries that occurred within 3 years prior to the study drug administration
- ➢ PTx (parathyroidectomy) or PEIT (percutaneous ethanol injection therapy)
- ➢ Any known history of allergy / drug sensitivity

5 STUDY DESIGN

5.1 Study Description

This study is an open-label, prospective, long term observational Phase IV study to assess the safety and efficacy of ferric citrate in subjects with ESRD on dialysis.

Subjects who meet the eligibility criteria and provided informed consent will be enrolled to the observational study for up to 13 months. No wash-out period will be required despite prior use of any oral phosphate binders.

Throughout the duration of the observation, study drug will be allowed for dose titration, targeting individual subject's serum phosphorus levels in between 3.5 and 5.5 mg/dL. Examinations to collect safety and efficacy measurements will be performed primarily according to routine hospital practice, except study drugs and additional tests that are specifically required to the study will be provided by the Sponsor.

The key measures collected for the patients will include, but not limited to, demography, medical history, physical examinations, vital signs, 12-lead electrocardiograms (ECG), clinical laboratory tests and prior/concomitant medications use for the evaluation of adverse events and treatment effectiveness throughout the observation period.

The end of the study is defined as the date of the last visit of the last subject in the study.

5.2 Planned Number of Subjects and Sites

It is planned to recruitment of 200 patients in order to complete at least 100 subjects at 5~10 centers in Taiwan.

6 SELECTION AND WITHDRAWAL OF SUBJECTS

6.1 Inclusion Criteria

To be eligible to participate in this study, all of the following criteria must be met:

1. Is ≥18 years of age on the day of signing informed consent or other age required by local regulation
2. Willing and able to provide written informed consent
3. ESRD patients who is undergoing hemodialysis 3 times per week and is considered necessary to receive medication for hyperphosphatemia by his/her treating physician
4. Serum ferritin <1000 ng/mL and transferrin saturation (TSAT) < 50% at the Enrollment Visit
5. Women of child-bearing potential (WOCBP [defined as women ≤ 50 years of age with a history of amenorrhea for < 12 months prior to study entry]) who is willing to use an effective form of contraception during study participation.**6.2 Exclusion Criteria**

Subjects are not permitted to enroll in the study if any of the following criteria is met:

1. Has any known contraindication to ferric citrate according to locally approved prescribing information, include but not limited to the following criteria:
   i. Is allergic to ferric citrate
   ii. Has hypophosphatemia
   iii. Has hemochromatosis or iron overload syndromes
   iv. Has active severe GI disorders
2. Has parathyroidectomy (PTx) or percutaneous ethanol injection therapy (PEIT) within 3 months prior to Enrollment Visit or serum calcium < 7 mg/dL at the Enrollment Visit
3. Has participated in another interventional study for any investigational agent or device within 30 days prior to enrollment
4. Is currently pregnant or breastfeeding

5. Other unstable medical condition or psychiatric conditions that is considered unsuitable for this study per Investigator's clinical judgment

6.3 Withdrawal Criteria

Subjects are free to withdraw from the study at any time, without prejudice to their continued care. A subject may be removed from the study at any time if any of the following events occurs:

- Subject request
- Treatment failure
- Investigator's discretion for the best interest of subject
- Intercurrent illness or medical event necessitating study drug discontinuation

If the subject withdraws, the final evaluations will be performed as completely as possible. A clear and concise reason for withdrawal should be recorded in the case report form (CRF). If the subject is withdrawn because of an AE, the subject should be followed until the AE resolves.

If a subject has serum phosphorous level >8.0 mg/dL in two consecutive monthly visits, not due to subject-reported non-compliance, the subject will be considered a treatment failure and will discontinue from the study.

7 STUDY MEDICATIONS

7.1 Initial Dose

All subjects will be instructed to take study drug intact with meals or immediately after meals and should not be opened or grounded.

Dose administration for the study drug will be at the discretion of the treating physicians and according to individual subject's clinical condition. The initial doses to be administered at study entry are suggested below:

- If the subject was on prior phosphate binders at a dosage equivalent to < 4.5g/day of calcium-based phosphate binders before entering the study, the suggested initial dose of study drug will be started from 3 g/day (2 capsules/meal, 6 capsules/ day).
- If the subject was on prior phosphate binders at a dosage equivalent to ≥ 4.5 g/day of calcium-based phosphate binders before entering the study, the suggested initial dose of study drug will be started from 4.5 g/day (3 capsules/meal, 9 capsules/ day).

7.2 Dose Adjustments

The dose will be adjusted to meet target goal for serum phosphorus of 3.5 to 5.5 mg/dL with a maximum dose of 12 g/ day. The suggested dose adjustments as outlined are to be considered as guidance for dose administration throughout the observation period, instead of mandated procedures. Dose may be adjusted by 1-2 g/day on a scheduled or unscheduled visit under the Investigator's discretion. Reasons for dose adjustments, interruption or resuming of study treatment should be recorded in the CRF.

7.3 Drug Accountability

At each visit after study drug is dispensed, subjects must return all unused IP and empty IP containers. Drug accountability must be done in the subject's presence in order to obtain explanations regarding discrepancies in compliance with the dosing regimen. Drug accountability must be recorded on the Drug Accountability form.

All returned trial medication will be assessed in order to monitor the subject's compliance with the medication schedule. All findings will be documented. If a subject was found to be persistently noncompliant (defined as ≤75% or ≥125% compliant with dosing schedule), Sponsor, in conjunction with Investigator, will decide whether the subject shall be withdrawn from the trial.

7.4 Prior and concomitant medication / treatments

All medication taken/treatments obtained before study start (initiated before study start) is termed prior medication/treatments.

Specific prior medication/treatments, including oral phosphate binders, ESA, iron preparation, aluminum containing medication, Vitamin D & calcium supplements, anti-osteoporotic medications, or other drugs that affect serum phosphorus level, during the past 3 months prior to study enrollment which meet the criteria below are considered relevant to the study indication and should be collected.

Any concomitant medication taken in addition to the study treatments and is considered relevant to influence serum phosphorous and/or iron levels should be collected throughout the study.

Other concomitant medications taken in addition to the study treatments which not fall into the table of specific medications, only minimal information will be collected throughout the study.

7.5 Prohibitions and Precautions

Subjects are prohibited to participate in clinical trial of other interventional drug or device throughout the study period.

Following precautions should be taken during the study:

- Subjects should be instructed to avoid taking ferric citrate concurrently with Aluminum-containing drugs.
- When using oral medications where a reduction in the bioavailability of the drug may cause clinically significant effect on its safety or efficacy, consider to administer the drug 2 hours before or after taking ferric citrate.
- The safety information of ferric citrate for pregnant and breastfeeding women has not been established. Female subjects of child-bearing potential are encouraged to take appropriate contraceptive procedures to avoid pregnancy during the study period.
- Caution should be taken to assess patients' overall condition if prescribing ferric citrate to patients with ferritin >800 ng/mL or TSAT >50%. Discontinue using of iron therapy if the symptom of iron overload is observed.

7.6 Blinding

This is an open-label, single arm study. No blind setting will be applied in this study.

8 STUDY PROCEDURES BY VISIT

8.1 Visit 1: Enrollment and Baseline Visit

Procedures are to be performed at Visit 1 including obtain signed Informed Consent Form from the subject, confirm eligibility, assign subject identifier, obtain demographic data, record CKD and other medical history, record Prior/Concomitant medications, perform physical examinations and obtain vital signs, perform baseline clinical laboratory tests, and study drug dispensing

8.2 Visit 2 to Visit 12 and Visit 14

The monthly visits will follow individual Investigator's routine clinical practice for each subject. However, it is suggested the monthly visits to be scheduled at regular intervals throughout the study period, e.g. approximately every 30 days.

Procedures are to be performed from Visit 2 through Visit 12, and the follow-up visit 14 including record concomitant medications, perform physical examinations and obtain vital signs, check for

adverse events, perform clinical laboratory test results, accountability on returned drugs and compliance check, and study drug dispensing

8.3 Visit 13: End-of-Treatment /Early Termination Visit

Procedures are to be performed at EOT including record concomitant medications, perform physical examinations and obtain vital signs, check for adverse events, perform clinical laboratory tests, and return all study drugs and empty containers

9 STATISTICS

A description of statistical methods is presented below and will be described in more detail in the Statistical Analysis Plan (SAP).

9.1 Determination of Sample Size

Since this is a single-arm observational study, and the major purpose is to collect long-term safety and efficacy of ferric citrate, the sample size of 200 subjects is driven by a post-approval commitment to the regulatory authority. Safety profile for a minimum drug exposure in 100 evaluable patients for 6 months and 1 year are required by the Taiwan regulatory authority. Taking reference from previous long-term extension study, total of 168 subjects were dosed in the 307 study and 125 subjects (74%) completed the 48 weeks treatment. Enrollment of 200 subjects in the present study should be sufficient to collect the long-term safety profile for 1 year in at least 100 patients.

9.2 Definition of Analysis Sets

Subjects will be categorized into the following populations to meet various study purposes.

Safety population: all subjects who are exposed to at least one dose of the study medication.

Full analysis set (FAS): the FAS population is defined as the subjects who satisfy all eligibility criteria, have taken at least one dose of study medication, and have at least one post treatment evaluation for efficacy.
Safety assessment will be determined on safety population. Baseline and demographic characteristics analyses will be performed on the safety population. Efficacy assessment will be performed on both safety and FAS populations, where efficacy conclusion will be made on FAS. For exploratory endpoints, descriptive statistics will be performed on both safety and FAS populations.

9.3 General Statistical Considerations

This is an observational study, all analysis will be performed based on descriptive statistics. No hypothesis testing will be performed.

Descriptive statistics such as mean, standard deviation, median, minimum and maximum values will be presented for the continuous variables and the number of patients and percentage will be presented for the categorical variables.

All therapies documented will be coded using the World Health Organization – Drug Dictionary (WHO-DD). Medical history, any diseases and AEs will be coded using the latest MedDRA version. The SAS® package (SAS® Institute Inc., USA, and Version 9.2 or later) will be used for statistical evaluation.

9.4 Analysis of endpoint

Safety assessment: This to assess the safety profiles from baseline to the EOT. Safety profiles will only be assessed by monitoring and recording the incidence of treatment-emergent adverse events

(TEAEs) and serious TEAEs, whether related to study treatment or not, as determined by changes in clinical laboratory tests (i.e., hematological tests and biochemical tests, including iron panels), physical examinations, vital signs, standard 12-lead electrocardiogram and any other untoward medical events during the study period. All subjects who receive at least one dose of study medication will be evaluated for safety.
TEAEs are defined as adverse events with an onset date on or after the first dose of study treatment. AEs with missing onset dates will be assumed to be treatment-emergent. Incidence of TEAEs will be summarized by the number, percentage, severity, and suspected relationship to study drug according to system organ class and preferred term using the MedDRA coding system.

Efficacy assessment: This is to describe the effectiveness of ferric citrate for the control of serum phosphorus concentration and achievement rate of target phosphorus level throughout 12 months of treatment. Treated patients who had an efficacy assessment at least once will be included in the full analysis set.

- Time-course changes of serum phosphorus levels from baseline to the end-of-treatment will be summarize according to each available observation visits. Continuous variables will be presented as mean ± standard deviation or median with interquartile range, as appropriate.
- Proportion of subjects achieved the target range of serum phosphorus (≥3.5 mg/dL and ≤ 5.5 mg/dL) from baseline to the end-of-treatment will be tabulated or plotted, as appropriate.

Exploratory endpoint: The following parameters will be explored:

- Time-course change of serum calcium and intact plasma parathyroid hormone (iPTH): Serum calcium and iPTH will be summarized descriptively as observed values at baseline, at every observation point, and at EOT. Changes from baseline will be descriptively summarized at completion of Visit 4 (Month 3), Visit 7 (Month 6), at Visit 13 (Month 12), and at EOT (including early termination).
- Time-course changes of iron-parameters from baseline to EOT: Serum iron, ferritin, TSAT, and TIBC will be summarized descriptively as observed values at baseline, every observation point, and EOT. Changes from baseline will be descriptively summarized at completion of Visit 4 (Month 3), Visit 7 (Month 6), at Visit 13 (Month 12), and at EOT (including early termination).
- Time-course changes of hemoglobin from baseline to EOT: Hemoglobin will be summarized descriptively as observed values at baseline, every observation point, and EOT. Changes from baseline will be descriptively summarized at completion of Visit 4 (Month 3), Visit 7 (Month 6), at Visit 13 (Month 12), and at EOT (including early termination).
- Changes in dose of IV iron and ESA from baseline to EOT: Dose of ESAs and intravenous iron preparations will be summarized descriptively at baseline, every observation point, and EOT.
- Treatment adherence: Dose of study drug dispensed and actually taken between each visit will be recorded. Proportion of the prescribed dose versus the dose actually taken at each time point will be calculated and summarized as percentages. Accumulated dose of individual subjects will be summarized by the end of treatment or at early termination.

Details will be specified in the statistical analysis plan (SAP).

9.7 Handling of Missing Data

Missing observations will not be imputed in the analysis. All statistics will be performed on available actual data.

10 ASSESSMENT OF SAFETY

10.1 Adverse Events

An adverse event (AE) is any untoward medical occurrence in a patient or clinical investigation in which a subject is administered with a pharmaceutical product which does not necessarily have a causal relationship with this treatment. An AE can therefore be any unfavorable and unintended sign (including an abnormal laboratory finding), symptom, or disease temporally associated with the use of a medicinal (investigational) product, whether or not related to the medicinal (investigational) product. Any worsening (i.e., any clinical significant adverse change in frequency and /or intensity) of a preexisting condition, which is temporally associated with the use of the investigational product, is also an AE.

To ensure complete safety data collection, all AEs occurring after signing the Informed Consent form, including any pre-treatment and post-treatment periods required by the protocol, must be recorded in the CRF even if no investigational product was taken but specific study procedures were conducted. Signs or symptoms of the condition/disease for which the investigational product is being studied should be recorded as AEs only if their nature changes significantly or their frequency or intensity increases in a clinically significant manner as compared to the clinical profile known to the Investigator from the subject's history or the Baseline Period.

Adverse events will be collected by means of a standard question, i.e., "Have you had any health problems since the previous visit?" This question will be asked to the subject at every time point after the initial dose of study drug is dispensed. When recording an AE, Investigator is strongly encouraged to use the overall diagnosis or syndrome using standard medical terminology, rather than recording individual symptoms or signs. The CRF and source documents should be consistent. Any discrepancies between the subject's own words on his/her own records (e.g., diary card) and the corresponding medical terminology should be clarified in the source documentation.

An AE should be followed until it has resolved, has a stable sequelae, the Investigator determines that it is no longer clinically significant, or the subject is lost to follow-up. If an AE is still ongoing at the end of the study for a subject, follow-up should be provided until resolution/stable level of sequelae, the Investigator no longer deems that it is clinically significant, or until the subject is lost to follow-up. If no follow-up is provided, the Investigator must provide a justification. The follow-up should be continued for approximately 1 month after the subject has discontinued their IP.

10.2 Serious Adverse Events

Once it is stated that a subject experienced an AE, the seriousness of the AE must be determined. An SAE must meet 1 or more of the following criteria:

- Results in death: This serious criterion applies if the subject's death is a direct outcome of a reported AE.
- Is a life-threatening experience: Any adverse event that places the subject, in view of the PI, at immediate risk of death from the adverse event as it occurred. It does not apply if an AE hypothetically might have caused death if it were more severe.
- Requires or prolongs in-patient hospitalization: This serious criterion applies if the reported AE requires at least a 24-hour inpatient hospitalization or, if in the opinion of the Investigator, it prolongs an existing hospitalization. A hospitalization for an elective procedure or a routinely scheduled treatment is not an SAE by this criterion because a "procedure" or a "treatment" is not an untoward medical occurrence.
- Causes persistent or significant disability/incapability: This serious criterion applies if the "disability" caused by the reported AE results in a substantial disruption of the subject's ability to carry out normal life functions.

- Results in congenital anomaly/birth defect: This serious criterion applies if a subject exposed to a medicinal (investigational) product gives birth to a child with congenital anomaly or birth defect.
- Is an important medical event: Any adverse event that, based upon appropriate medical judgment, may jeopardize the subject and may require medical or surgical intervention to prevent one of the outcomes listed above.

Any SAE, whether or not related to the study drug, occurring after the patients has provided consent and until at least 30 days after the patient has stopped study treatment must be reported to Sponsor or its their representative within 24 hours of being aware of its occurrence. Any SAEs experienced after this 30 days period should only be reported to Sponsor if the Investigator suspects a causal relationship to the study treatment.

All SAEs must be reported within 24 hours of their occurrence. This can be done by faxing a completed SAE Fax Cover Sheet and SAE CRFs or direct telephone communication to the Sponsor and their representative. A completed SAE Fax Cover Sheet and SAE CRF should follow all telephone reports within 24 hours.
Upon receipt of the SAE Form, Sponsor will perform an assessment of expectedness of the reported SAE. The assessment of the expectedness of the SAE is based on the latest updated Investigators' brochure.

An SAE should be followed until it has resolved, has a stable sequelae, the Investigator determines that it is no longer clinically significant, or the subject is lost to follow-up. Information on SAEs obtained after clinical database lock will be captured through the GCSP database without limitation of time.

10.3 Pregnancy
Although not considered an AE, it is the responsibility of Investigator or their designees to report any pregnancy in a subject (spontaneously reported to them) that occurs during the study. If a subject becomes pregnant after the first intake of any IP, Sponsor's Clinical Safety or Pharmacovigilance department should be informed within 24 hours of learning of its occurrence. As soon as the pregnancy is known and the following should be conducted:
- The subject should immediately stop the intake of the study drug.
- The subject should return for an EOT visit.

The health of the child must be followed for 30 days after birth for any significant medical issues. In certain circumstances, Sponsor may request that follow-up is continued for a period longer than 30 days. A pregnancy becomes a SAE if miscarriage, abortion, or anomaly/birth defect of the child. Those SAEs must be additionally reported using the SAE Report Form.

11 STUDY MANAGEMENT AND ADMINISTRATION
11.1 Adherence to Protocol
The Investigator should not deviate from the protocol. In medical emergencies, the Investigator may use his/her medical judgment and may remove a study participant from immediate hazard before notifying Sponsor (or its representative) and the IRB/IEC in writing regarding the type of emergency and the course of action taken.

11.2 Monitoring
Sponsor (or designee) will monitor the study to meet the sponsor's monitoring Standard Operating Procedures (SOPs), ICH-GCP guideline, and applicable regulatory requirements, and to ensure that

study initiation, conduct, and closure are adequate. Monitoring of the study may be delegated by Sponsor to a contract research organization (CRO) or a contract monitor.

11.3 Data Handling

The Investigator is responsible for prompt reporting of accurate, complete, and legible data in the CRFs and in all required reports. Any change or correction to the CRF should be dated, initiated, and explained (if necessary) and should not obscure the original entry. Use of correction fluid is not permitted. Corrections made after the Investigator's review and signature of the completed CRF will be resigned and dated by the Investigator. Case report forms/external electronic data will be entered/loaded in a validated electronic database using a clinical data management system (CDMS). Computerized data cleaning checks will be used in addition to manual review to check for discrepancies and to ensure consistency of the data. Case report form data are entered into the clinical database using independent, double-data entry, with the exception of comment fields, which are verified by a second person. In the event that the study is performed using RDC, the data are entered into the electronic CRFs once and are subsequently verified. An electronic audit trail system will be maintained within the CDMS to track all data changes in the database once the data has been saved initially into the system or electronically loaded. Regular backups of the electronic data will be performed. The Investigator will maintain adequate records for the study including CRFs, medical records, laboratory results, Informed Consent documents, drug dispensing and disposition records, safety reports, information regarding participants who discontinued, and other pertinent data.

11.4 Good Clinical Practice

Noncompliance with the protocol, ICH/GCP, or local regulatory requirements by the Investigator, institution, institution staff, or designees of the sponsor will lead to prompt action by Sponsor to secure compliance. Continued noncompliance may result in the termination of the site's involvement in the study.

12 ETHICS AND REGULATORY REQUIREMENTS

12.1 Informed Consent

Subject's informed consent must be obtained and documented in accordance with local regulations, ICH-GCP requirements, and the ethical principles that have their origin in the principles of the Declaration of Helsinki. Prior to obtaining informed consent, information should be given in a language and at a level of complexity understandable to the subject in both oral and written form by the Investigator (or designee). Each subject will have the opportunity to discuss the study and its alternatives with the Investigator. Prior to participation in the study, the written Informed Consent form should be signed and personally dated by the subject, or his/her legal representative, and by the person who conducted the informed consent discussion (Investigator [or designee]). The subject or his/her legal representative must receive a copy of the signed and dated Informed Consent form. As part of the consent process, each subject must consent to direct access to his/her medical records for study-related monitoring, auditing, IRB/IEC review, and regulatory inspection.

12.2 Institutional Review Boards and Independent Ethics Committees

The study will be conducted under the auspices of an IRB/IEC, as defined in local regulations, ICH-GCP, and in accordance with the ethical principles that have their origin in the Declaration of Helsinki. The Investigator/ Sponsor will ensure that an appropriately constituted IRB/IEC that complies with the requirements of the current ICH-GCP version or applicable country-specific regulations will be responsible for the initial and continuing review and approval of the clinical study. Prior to initiation of the study, the Investigator/ Sponsor will forward copies of the protocol, Informed Consent form, Investigator's Brochure, Investigator's curriculum vitae (if applicable),

advertisement (if applicable), and all other subject-related documents to be used for the study to the IRB/IEC for its review and approval. Before initiating a study, the Investigator will have written and dated full approval from the responsible IRB/IEC for the protocol. The Investigator will also promptly report to the IRB/IEC all changes in the study, all unanticipated problems involving risks to human subjects or others, and any protocol deviations, to eliminate immediate hazards to subjects. The Investigator will not make any changes in the study or study conduct without IRB/IEC approval, except where necessary to eliminate apparent immediate hazards to the subjects. For minor changes to a previously approved protocol during the period covered by the original approval, it may be possible for the Investigator to obtain an expedited review by the IRB/IEC as allowed. As part of the IRB/IEC requirements for continuing review of approved studies, the Investigator will be responsible for submitting periodic progress reports to the IRB/IEC (based on the Committee's requirements), at intervals appropriate to the degree of subject risk involved but no less than once per year. The Investigator should provide a final report to the IRB/IEC following study completion. Sponsor (or its representative) will communicate safety information to the appropriate regulatory authorities and all active Investigators in accordance with applicable regulatory requirements. The appropriate IRB/IEC will also be informed by the Investigator or the sponsor, as specified by the applicable regulatory requirements in each concerned country. Where applicable, Investigators are to provide the sponsor (or its representative) with evidence of such IRB/IEC notification.

12.3 Subject Confidentiality

Sponsor staff (or designee) will affirm and uphold the subject's confidentiality. Throughout this study, all data forwarded to Sponsor (or designee) will be identified only by the subject number assigned at Screening. The Investigator agrees that representatives of Sponsor, its designee, representatives of the relevant IRB/IEC, or representatives of regulatory authorities will be allowed to review that portion of the subject's primary medical records that directly concerns this study (including, but not limited to, laboratory test result reports, ECG reports, admission/discharge summaries for hospital admissions occurring during a subject's study participation, and autopsy reports for deaths occurring during the study).

12.4 Protocol Amendments

Protocol changes may affect the legal and ethical status of the study and may also affect the statistical evaluations of sample size and the likelihood of the study fulfilling its primary objective.Significant changes to the protocol will only be made as an amendment to the protocol and must be approved by Sponsor, the IRB/IEC, and the regulatory authorities (if required), prior to being implemented.

13 FINANCE, INSURANCE, AND PUBLICATION

Insurance coverage will be handled according to local requirements. Examinations to collect safety and efficacy measurements will be performed primarily according to routine hospital practice. Study drugs and grants for essential examinations which are study mandatory tests, i.e. iron panels, intact-PTH, Aluminum, ECG, or those which not following hospital's routine examinations will be provided by the Sponsor. Finance, insurance, and publication rights are addressed in the Investigator and/or CRO agreements as applicable.

14 REFERENCES

1. Slatopolsky, E., A. Brown, and A. Dusso, *Pathogenesis of secondary hyperparathyroidism.* Kidney Int Suppl, 1999. **73**: p. S14-9.

2. Lau, K., *Phosphate excess and progressive renal failure: the precipitation-calcification hypothesis.* Kidney Int, 1989. **36**(5): p. 918-37.
3. Haut, L.L., et al., *Renal toxicity of phosphate in rats.* Kidney Int, 1980. **17**(6): p. 722-31.
4. Karlinsky, M.L., et al., *Preservation of renal function in experimental glomerulonephritis.* Kidney Int, 1980. **17**(3): p. 293-302.
5. Klein, G.L., *Aluminum in parenteral solutions revisited--again.* Am J Clin Nutr, 1995. **61**(3): p. 449-56.
6. Klein, G.L., *Nutritional aspects of aluminium toxicity.* Nutr Res Rev, 1990. **3**(1): p. 117-41.
7. Klein, G.L., et al., *Aluminum as a factor in the bone disease of long-term parenteral nutrition.* Trans Assoc Am Physicians, 1982. **95**: p. 155-64.
8. McDonald, S.J., E.M. Clarkson, and H.E. Dewardener, *The Effect of a Large Intake of Calcium Citrate in Normal Subjects and Patients with Chronic Renal Failure.* Clin Sci, 1964. **26**: p. 27-39.
9. Clarkson, E.M., S.J. McDonald, and H.E. De Wardener, *The effect of a high intake of calcium carbonate in normal subjects and patients with chronic renal failure.* Clin Sci, 1966. **30**(3): p. 425-38.
10. Hsu, C.H., *Are we mismanaging calcium and phosphate metabolism in renal failure?* Am J Kidney Dis, 1997. **29**(4): p. 641-9.
11. Clarkson, E.M., et al., *The effect of a high intake of calcium and phosphate in normal subjects and patients with chronic renal failure.* Clin Sci, 1970. **39**(6): p. 693-704.
12. Clarkson, E.M., et al., *Net intestinal absorption of calcium in patients with chronic renal failure.* Kidney Int, 1973. **3**(4): p. 258-63.
13. *Renagel® Product Information*. Genzyme Corporation, Cambridge, MA: Available at http://products.sanofi.us/Renvela/Renvela.html (as of May 19, 2015).
14. Yang, W.C., et al., *An open-label, crossover study of a new phosphate-binding agent in haemodialysis patients: ferric citrate.* Nephrol Dial Transplant, 2002. **17**(2): p. 265-70.
15. Sinsakul, M., et al., *The safety and tolerability of ferric citrate as a phosphate binder in dialysis patients.* Nephron Clin Pract, 2012. **121**(1-2): p. c25-9.
